# Early visual evoked potentials are modulated by eye position in humans induced by whole body rotations

**DOI:** 10.1186/1471-2202-5-35

**Published:** 2004-09-19

**Authors:** Frédéric Andersson, Olivier Etard, Pierre Denise, Laurent Petit

**Affiliations:** 1 Groupe d'Imagerie Neurofonctionnelle (GIN) UMR6194, CNRS-CEA-University of Caen and Paris 5, GIP CYCERON, BP5229, Bd. Becquerel, 14074 Caen Cedex, France; 2 Service d'Explorations du Système Nerveux, CHU Caen, 14033 Caen Cedex, France

## Abstract

**Background:**

To reach and grasp an object in space on the basis of its image cast on the retina requires different coordinate transformations that take into account gaze and limb positioning. Eye position in the orbit influences the image's conversion from retinotopic (eye-centered) coordinates to an egocentric frame necessary for guiding action. Neuroimaging studies have revealed eye position-dependent activity in extrastriate visual, parietal and frontal areas that is along the visuo-motor pathway. At the earliest vision stage, the role of the primary visual area (V1) in this process remains unclear. We used an experimental design based on pattern-onset visual evoked potentials (VEP) recordings to study the effect of eye position on V1 activity in humans.

**Results:**

We showed that the amplitude of the initial C1 component of VEP, acknowledged to originate in V1, was modulated by the eye position. We also established that putative spontaneous small saccades related to eccentric fixation, as well as retinal disparity cannot explain the effects of changing C1 amplitude of VEP in the present study.

**Conclusions:**

The present modulation of the early component of VEP suggests an eye position-dependent activity of the human primary visual area. Our findings also evidence that cortical processes combine information about the position of the stimulus on the retinae with information about the location of the eyes in their orbit as early as the stage of primary visual area.

## Background

In humans, goal-directed movements to an object in space are improved by foveal vision, namely by gaze anchoring on the object [1]. Since motor and visual information are encoded in different reference frames, accurate reaching and grasping movements in space require ongoing registration and coordinate transformation of visual percepts with gaze and limb positioning. One essential transformation is to convert the retinal image from eye-centered coordinates in a target location with respect to the head by taking into account the position of the eyes in the orbit [2]. Single-cell recordings in monkeys revealed that the neural substrate of the visual-to-motor coordinate transformations is a change in the visual or motor response properties according to gaze position in extrastriate visual, parietal, oculomotor, premotor and motor areas [for review see [3,4]]. Functional magnetic resonance imaging (fMRI) studies have localized human homologues of such monkey areas and have showed that eye position signals modulate activity of extrastriate visual areas [5] and the parieto-frontal network related to hand-arm movements [6,7]. Less is known at the earliest stage of visual processing, namely in the primary visual area (V1). Previous electrophysiological [8-10] and modeling [11] studies described eye position dependent activity in V1 neurons to a lesser extent than that reported in parietal and premotor cortex but consistent with the idea that both retinal and eye position signals may also converge at early vision stage. However, the direct influence of eye position on V1-related activity in humans has not been investigated. Consequently, we used a specific experimental design based on pattern-onset visual evoked potentials (VEP) recordings to study the effect of eye position exclusively on V1 activity in humans. Thus, we focused our investigation on the first major VEP component C1, obtained using pattern-onset stimulation, because its distribution over the scalp and its retinotopic properties indicate an origin from the calcarine fissure that is V1. This issue has been the conclusion of all previous studies over the past 10 years regarding the cortical visual areas that generate the early components of pattern-onset VEP [for review see [12]]. The most recent reports even demonstrated in individual subjects a close anatomical correspondence between modelled dipoles for the C1 component and sites of activation in the calcarine fissure obtained in fMRI in response to the same visual stimuli [12-14]. The purpose of the present report was therefore to investigate the eye position-dependent activity of V1 in humans by characterizing the early C1 component of VEP and testing its properties at different eye positions.

## Results and discussion

**Figure 1:**
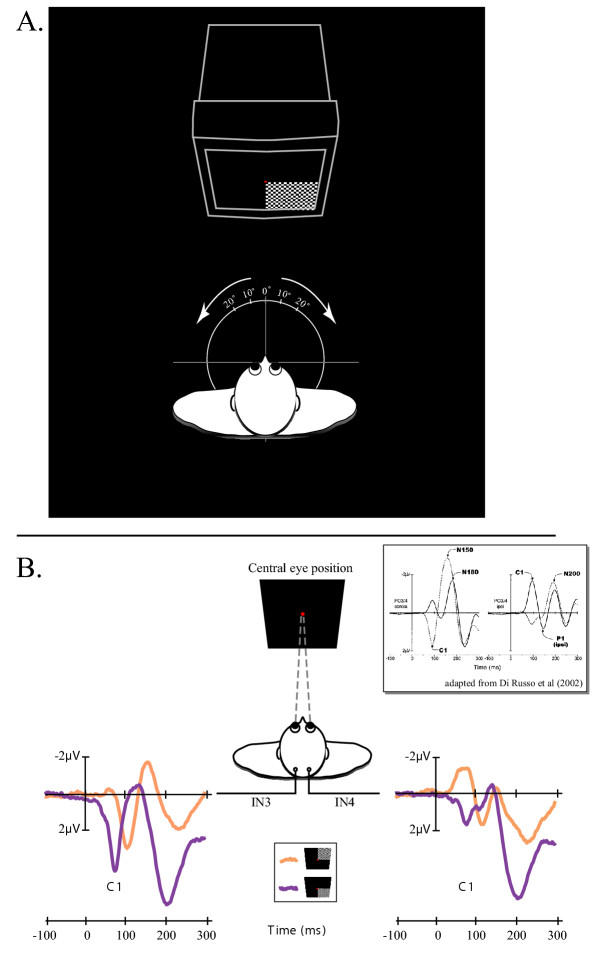
A. Experimental design. See methods for details. B. Polarity inversion of the C1 component observed on the grand averaged VEP over the 20 subjects and in response to stimuli in the upper (in orange) and lower (in purple) quadrants of the right visual fields. The present polarity inversion was observed on both IN3 and IN4 intermediate occipital sites. The box adapted from Di Russo et al (2002) represents the polarity inversion of the C1 components on the grand average VEPs in response to upper (solid line) and lower (dashed line) hemifield stimuli. In this study, waveforms were collapsed across VEPs to left and right hemifield stimuli and were plotted separately for scalp sites contralateral (left) and ipsilateral (right) to the side of the stimulation. The polarity inversion was observed prominently on occipito-parietal sites PO3/4 using a 10–10-system montage.

The C1 component reverses classically in polarity for upper *vs. *lower visual field stimulation [for review see [12]]. Consequently, we first characterized the C1 component over the 20 subjects by observing its polarity inversion for a central eye position. Figure 1B illustrates the representative polarity inversion of the C1 component on the grand averaged of VEP over the 20 subjects and in response to stimuli in the upper and lower quadrants of the right visual fields. Equivalent VEP and C1 polarity inversion were obtained in response to stimuli in the upper and lower quadrants of the left visual fields. The polarity inversion of the C1 components on the grand average VEPs in response to upper and lower hemifield stimuli were described previously for occipito-parietal sites using a 10–20 system montage with 62 scalp sites (see box included in Figure 1). It is noteworthy that a similar polarity inversion was measured in our study using only two occipital intermediate sites (IN3 and IN4) of the modified 10–20-system montage (see Methods for details).

Once the C1 component was characterized for both upper and lower quadrant visual fields, the effect of eye position on C1 amplitude was measured only for both left and right lower quadrants visual field that is for the most salient C1 component which we observed.

Mean peak latencies of C1 was calculated in each subject in response to both left and right lower visual quadrants and for both IN3 and IN4 recording sites. They ranged between 94.8 ms and 98.0 ms that are consistent with the C1 latency range previously observed in numerous studies [12,15]. For each subject, the C1 amplitude for five different eye positions (0°, 10° and 20° both left- and rightward) was then measured at these mean latencies for each lower quadrant and each recording site and, with respect to a 80 ms pre-stimulus baseline.

**Figure 2:**
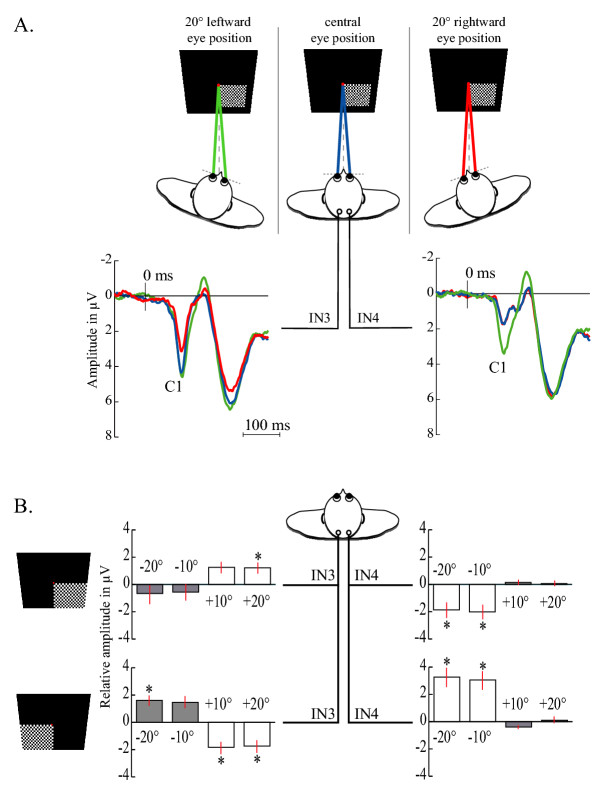
A. Example of modulation of the C1 amplitude observed for both left- and right-ocular deviations of 20°. The grand averaged VEP over the 20 subjects and in response to stimuli in the lower quadrants of the right visual field is presented at each lateral site (IN3, IN4) for central eye position (blue) and, 20° leftward (green) and 20° rightward (red) eye positions. B. Comparison of the difference of C1 amplitudes between each deviated eye position and the central eye position for both left and right lower quadrant field. For each subject, the C1 amplitude calculated for each eye position was subtracted from the C1 amplitude measured for the central eye position. Vertical bars represent the standard error of the mean.

We observed that checkerboard presented in the lower visual field elicited VEPs with modulated amplitudes of the C1 component in respect of eye position. Grand averaged VEPs over the 20 subjects in response to flashed stimuli in the right lower quadrant of the visual field for three different eye positions (0° and 20° both left- and rightward) are shown in Figure 2A. It is noteworthy that the eye position-dependent modulation of C1 amplitude was observed at the IN3 recording site for a 20° rightward eye position (Figure 2A, red trace) and at the IN4 recording site with a 20° leftward eye position (Figure 2A, green trace).

In other words, the eye position effect was observed at the occipital recording site contralateral to the direction of the eye deviation. Equivalent VEPs but reverse eye-position effects were obtained in response to left field stimuli. A one-way Friedman repeated measures analysis of variance was conducted for each recording site and for each visual stimulation revealing a significant main effect of eye position on the amplitude of the C1 component. Complete data obtained at both recording sites, for each lower visual field are shown in Figure 2B. We chose to represent these data using relative amplitude measures that are amplitude for deviated eye position subtracted by the amplitude for central eye position. It means that amplitude zero corresponds to the central eye position situation. A post-hoc Dunnett's test (p < 0.05) using 0° as reference showed that the amplitude of the C1 component measured contralaterally to the deviated eye position was significantly different from 0° except for one situation (Figure 2B, white histograms). Conversely, the amplitude of the C1 component recorded ipsilaterally to the deviated eye position was not significantly different from 0° excepted for one situation (Figure 2B, grey histograms). Note that no parametric effect was observed for the C1 amplitude between 10° and 20° of eye eccentricity (Student's t-test, p < 0.05).

The overall result of the present study revealed that the amplitude of first major component of VEP elicited by checkerboard (C1) is modulated by the eye position. The previous data linking the C1 component to a striate cortex generator, namely V1 activity (see introduction), led us to suggest that eye position influences the earliest cortical stage of visual processing.

One may first suggest that the present results may be explained by the difficulty of maintaining eccentric fixation, which may have altered the pattern of fixational eye movements, such as microsaccades [fast, conjugate jerks, smaller than 1/3°, see [16]]. Recent studies have shown that microsaccades modulated neural activity in V1 [17,18], but to our knowledge no study has examined the effect of maintaining eccentric fixation on the occurrence of microsaccades. In the present study, we were able to track spontaneous saccadic eye movements superior to 1°. A one-way Friedman repeated measures analysis of variance for each visual stimulation revealed no significant main effect of eye position on the amplitude (p = 0.35) and on the frequency (p = 0.45) of saccades superior to 1°. We cannot rule out quantitatively a possible role for microsaccades in the eye position-dependent V1 activity. Regardless of such putative effects, however, one may argue that in case of an increase of the number of microsaccades related to eccentric fixation, a similar effect in terms of magnitude would be observed for both left- and rightward deviation. This was not the case in the present study.

**Figure 3:**
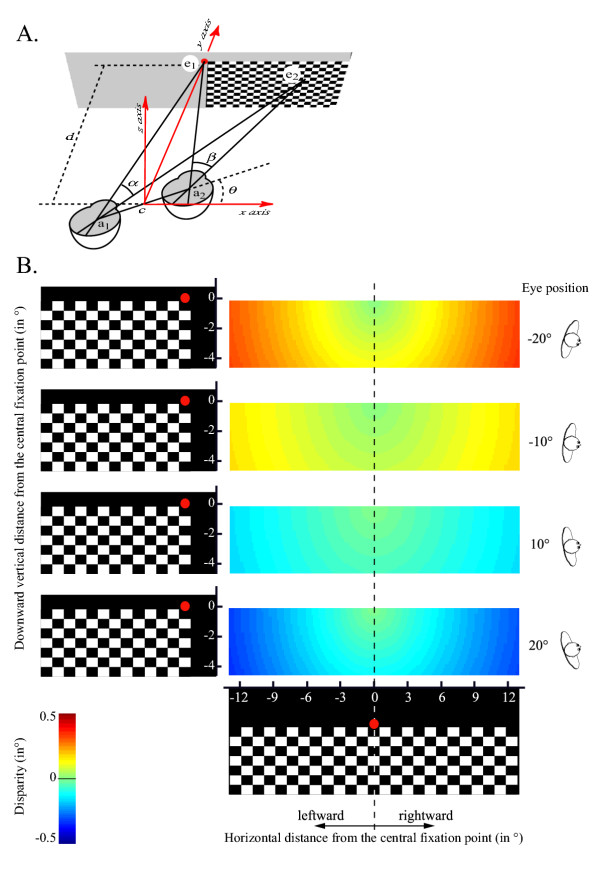
A. Schematic experimental design. The cyclopean axis (c) is defined by the middle of both left and right eye rotation axis, a1 and a2 respectively. Both a1 and a2 also designates the interocular distance (a). θ corresponds to the subject's head position relative to the screen axis. Along the horizontal axis crossing the screen, (e1) corresponds to the centre of the screen, namely the fixation point, and (e2) represents a given point on the screen. Both α and β correspond to the retinal angles of the segment [e1e2] seen by the left and the right eye, respectively. The distance (δ) between the middle of the cyclopean axis (c) and the fixation point (ε1), in other words the distance between the screen and the subject was 1 m in the present study. B. Plot of the magnitude of disparity for each point of the checkerboard and for each eye's deviation [-20°, -10°, 10°, 20°] normalized by the disparity calculated for the central eye position (θ = 0°, see methods for details of the calculation).

The question also arises if the effects of changing C1 amplitude may be due to oculomotor signals and/or retinal disparity that is to the difference in the position of the visual stimulus on each retina related to relative monitor distance. The absence of any parametric effect for the C1 amplitude between 10° and 20° of eye eccentricity may indicate that eye position effects are not due to the difference in the horizontal retinal disparity, but one may argue that a putative relationship between the horizontal disparity and the C1 amplitude is not linear. We evaluated the difference of both horizontal and vertical disparity between the different eye's deviations in our study (see Methods for details and Figure 3). Figure 3B shows that the difference between the magnitude of disparity for the central eye position and each deviated eye condition depends on both eye deviation and the distance of the visual stimulus from the fixation point. Interestingly, such a difference in terms of horizontal and vertical disparity does not depend on the stimulated quadrant visual field in the range of the present checkerboard's width. In other words, the magnitude of the relative disparity for a given distance from the fixation point and for a given deviated eye condition is similar for both left and right visual field stimulation. Since we observed that the C1 amplitude varied inversely in function of the hemifield stimulation (Figure 2), the eye-position effect observed on the C1 amplitude cannot be simply related to a horizontal and/or vertical disparity effect.

Both direct and indirect arguments also suggest that variations in the C1 amplitude are not due to attention. Firstly, the subjects were instructed to keep firmly visual attention on the fixation point suggesting that the potential degree of attention required fixating binocularly the red fixation dot was similar across the different deviated eye positions. Secondly, numerous recent studies gave impetus to an emerging view that V1 activity may be modulated by attention through delayed feedback signals (160–260 ms) from extrastriate and/or oculomotor structures while the initial C1 sensory response (50–90 ms) was not modulated by attention [13-15,19-22]. Our present findings, together with those aforementioned, allow considering that both eye position and attention-related signals may affect the early stage of visual processing in different manner. The former may comes from extraocular muscle afferents and/or corollary discharges while the later is considered as a late top-down process [14,20].

Finally, Trotter et al. (1999) have shown that an eye position signal (extraretinal signal) is involved in the neural modulation process dealing with the eye position-dependent visual response observed in area V1 of behaving monkeys. Oculomotor signals coming from extraocular muscle afferents and/or corollary discharges are considered as the substrate of such an eye position signal and have been previously described in V1 [23,24].

## Conclusions

The present results and previous works obtained from neural recordings in monkeys indicate that changes in eye position can modify response properties in V1 that is at the earliest cortical stage of visual processing. Among the visuo-motor processing allowing accurate reaching and grasping movements in space on the basis of the image seen by the retina, the primary visual area may be therefore one of the first cortical relay to convert the image in eye-centered coordinates into a target location by taking into account the position of the eyes in the orbit. It endorses some recent arguments pointing out that V1 could no longer be considered only in relation to the pattern of light falling on the retina but appears to be a cortical area in which contextual influences take place too [9].

## Methods

### Subjects

Twenty right-handed healthy volunteers with normal visual acuity (range age 19–29 years, 9 males) participated in VEP recordings after they provided their written informed consent. The study was approved by the Basse-Normandie ethics committee (Caen, France).

### Experimental design

The subjects were seated on a swivel armchair with their head stabilized with a headrest. An experimenter slowly rotated the armchair and locked it in one of the five different angles: 0°, 10° and 20° both left- and rightward from the center of the monitor leading to five different eye positions (Figure 1A). The vertical swivel axis passed through the base of the nose between both eyes. Such a passive vertical movement of rotation of the whole body stimulated only the horizontal semicircular canals of the vestibular system with a time constant around 15 sec [25]. The VEP recording was started at least 1 minute after the rotational movement in order to stay away from the influence of the passive whole body rotational movement of the subjects. The subjects had to fixate binocularly a red fixation dot continuously visible in the center of the display as stimulus was flashed in 1 of the 4 quadrants of the visual field. Upper quadrants were used only in the case of central eye position in order to observe the inversion of polarity of the C1 component that allowed its detection on VEP recordings. The stimulus consisted of a black and white rectangular checkerboard (12 × 9° of visual angle, 0.6 cycle.deg^-1 ^of spatial frequency) flashed against a black background (ISI = 500–1000 ms) and delivered by a visual stimulator (Nicolet, Madison, USA). The subjects were instructed to fixate continuously the fixation dot and to keep their attention on it, for each quadrant of the visual field so that the projection of the stimulus on the retina was equivalent whatever was the deviated eye position. The edges of the monitor and the space up to 1 meter around the monitor were masked with an opaque black sheet preventing any cue perception in the room except the flashing checkerboard.

### VEP recordings

With respect to the purpose of the present study, we recorded VEP from the scalp using the two occipital intermediate sites (IN3 and IN4) of the modified 10–20-system montage [26] with both left and right mastoids serving as reference. The VEP from each site was recorded (Vicking, Nicolet, Madison, USA) at a sampling rate of 2500 Hz (0.1–100 Hz of band-pass filter with a 50 Hz notch filter). Prior to averaging, artifact rejection was performed to discard epochs with eye blinks. A total of 200 non-rejected epochs was averaged for each recording. Both horizontal and vertical eye movements were also monitored in each subject and during all VEP recordings, with EOG electrodes placed around the orbit of the right eye. The EOG system had a resolution superior to 1° of visual angle. All EOG records were analyzed by computer, using a dedicated software (SAMO) [27] which detects saccadic components and quantifies the amplitude and frequency of the spontaneous saccadic eye movements.

### Measurement of the disparity

We evaluated the difference of disparity between the different eye's deviations (Figure 3). The difference in retinal angles (β - α) defines the magnitude of disparity, classically designated (η) [28]. Such a disparity depends on the position of the head's subject relative to the screen, designated (θ), on the interocular distance (a), and on the distance (d) between the middle of the cyclopean axis (c) and the fixation point (e1). We evaluated the magnitude of the disparity for any point (e2) of the checkerboard (Figure 3A).

Using the dot product, for the left eye, α can be expressed as following:

a1e1.a1e2 = |a1e1| |a1e2| cos(α)     (1)

Expressing of the coordinates of the vectors a1e1 and a1e2 in the canonical reference (*x,y,z*) centred on the cyclopean axis (c):

a1e1.a1e2 = a1e1_x _a1e2_x _+ a1e1_y _a1e2_y _+ a1e1_z _a1e2_z _    (2)

and combining both (1) and (2) led to:

cos(α) = (a/2 cos(θ))(e2_x_+a/2 cos(θ)) + (d +a/2sin(θ))^2 ^/ |a1e1| |a1e2|     (3)

Similarly, for the right eye, we obtained:

cos (β) = (-a/2cos(θ))(e2_x_-a/2cos(θ)) + (d -a/2sin(θ))^2 ^/ |a1e1| |a1e2|     (4)

Applying the numeric values defined in our study (d = 1 m, a = 8 cm), and combining both (3) and (4) allows to estimate the magnitude of the disparity (η = β-α) in function of the distance from the fixation point for each given eye's deviation. Therefore, we plotted the magnitude of disparity with each distance (e) and for each eye's deviation [-20°, -10°, 10°, 20°] normalized by the disparity calculated for the central eye position (θ = 0°, Figure 3B).

## List of abbreviations used

C1 : early component of visual evoked potential

fMRI : functional magnetic resonance imaging

IN3 and IN4 : occipital intermediate recording sites

ISI : inter-stimulus interval

V1 : primary visual area

VEP : visual evoked potential

## Authors' contributions

All authors designed the study. FA and OE carried out the experiments and generated the method for data analysis. OE performed the statistical analysis and generated the method for estimation of the magnitude of disparity. FA and LP wrote the first versions of the manuscript. All authors read, discussed and approved the final version of the manuscript.
